# Supplementary material for: Heat Stress in relation to Sleep Health among Farmers
Source: medRxiv. 2025 Jul 29:2025.07.28.25332331. Preprint. [Version 1] doi: 10.1101/2025.07.28.25332331 (PMC12324656; doi:10.1101/2025.07.28.25332331)
Supplement: 1 [file NIHPP2025.07.28.25332331V1-supplement-1.pdf]

medRxiv preprint doi: https://doi.org/10.1101/2025.07.28.25332331; this version posted July 29, 2025. The copyright holder for this preprint (which was not certified by peer review) is the author/funder, who has granted medRxiv a license to display the preprint in perpetuity.
This article is a US Government work. It is not subject to copyright under 17 USC 105 and is also made available for use under a CC0 license.

Supplemental materials

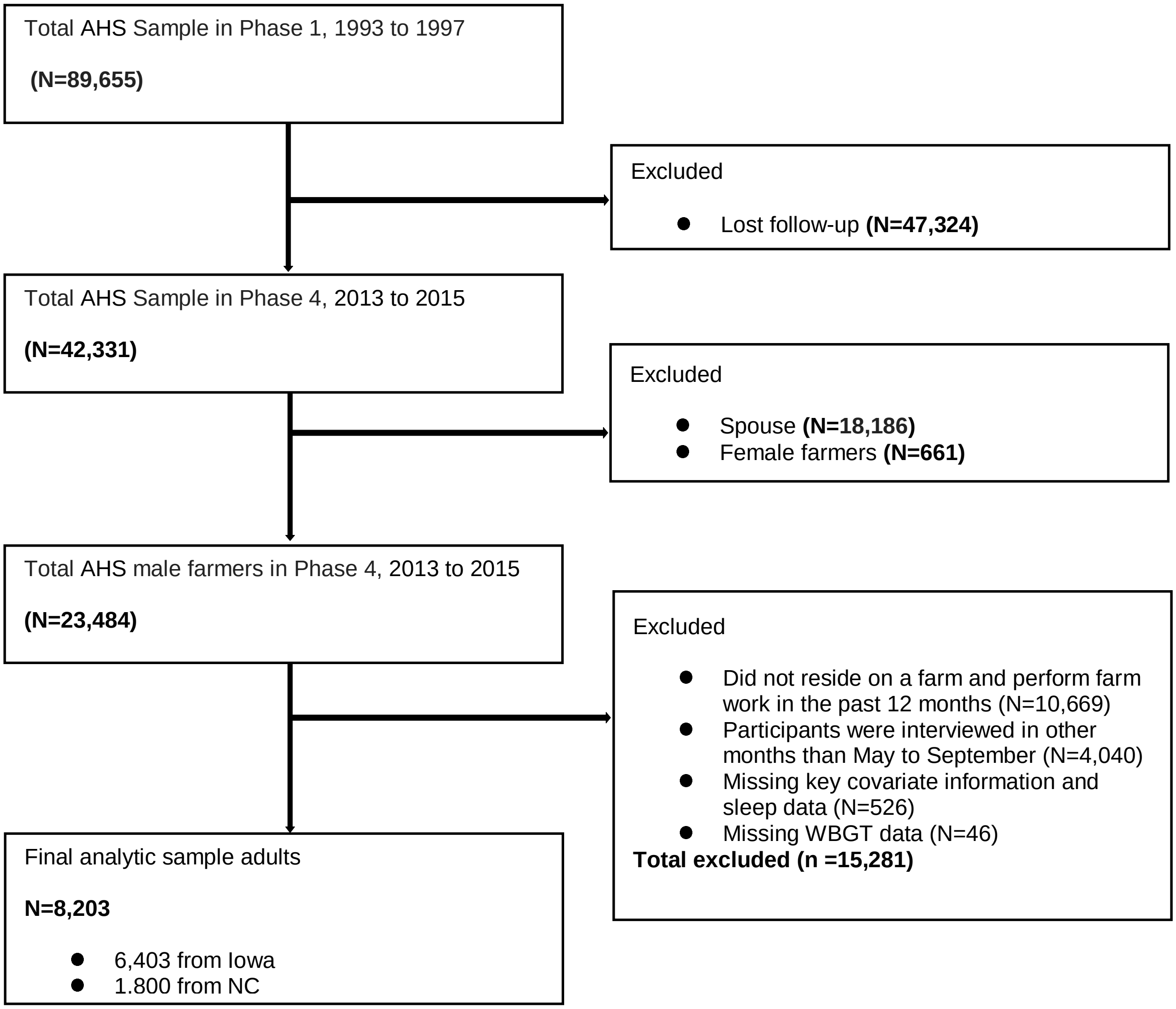

Figure S1. The flow chart of sample selection

medRxiv preprint doi: https://doi.org/10.1101/2025.07.28.25332331; this version posted July 29, 2025. The copyright holder for this preprint (which was not certified by peer review) is the author/funder, who has granted medRxiv a license to display the preprint in perpetuity. This article is a US Government work. It is not subject to copyright under 17 USC 105 and is also made available for use under a CC0 license.

Table S1. Basic characteristics among excluded and included participants

	**Included: n=8,203**	**Excluded: n=15,281**
**Sociodemographic characteristics**		
Age, mean (SD) [a]	63.0 (10.1)	68.3 (12.1)
Missing	0	0
Age categories		
<50	651 (7.9)	911 (6.0)
51-60	2612 (31.8)	3019 (19.8)
61-70	2762 (33.7)	4119 (27.0)
>70	2178 (26.6)	7232 (47.3)
Missing	0	0
Race and ethnicity [b, c]		
Non-Hispanic White	8132 (99.1)	14636 (95.8)
Other	71 (0.9)	355 (2.3)
Missing	0	290 (1.9)
Educational attainment [c]		
1-8 years	147 (1.8)	643 (4.2)
Some high school	198 (2.4)	761 (5.0)
High school graduate or higher	7858 (95.8)	13059 (85.5)
Missing	0	818 (5.4)
Marital status [d]		
Married/Cohabitating	7174 (87.5)	10803 (70.7)
Other	1029 (12.5)	2007 (13.1)
Missing	0	2471 (16.2)
Geographical region		
Iowa	6403 (78.1)	9461 (61.9)
NC	1800 (21.9)	5668 (37.1)
Missing	0	152 (1.0)
**Sleep outcomes**		
Sleep duration [e]		
≥7 hours	5104 (62.2)	7906 (51.7)
<7 hours	3099 (37.8)	4794 (31.4)
Missing	0	2581 (16.9)
Daytime sleepiness [f]		
<3 days/week	7539 (91.9)	11191 (73.2)
≥3 days/week	664 (8.1)	1399 (9.2)
Missing	0	2691 (17.6)
Daytime napping [g]		
No	4543 (55.4)	6572 (43.0)
Yes	3660 (44.6)	6130 (40.1)
Missing	0	2579 (16.9)
Napping duration [h]		

medRxiv preprint doi: https://doi.org/10.1101/2025.07.28.25332331; this version posted July 29, 2025. The copyright holder for this preprint (which was not certified by peer review) is the author/funder, who has granted medRxiv a license to display the preprint in perpetuity. This article is a US Government work. It is not subject to copyright under 17 USC 105 and is also made available for use under a CC0 license.

≥30 minutes	1401 (17.1)	3145 (20.6)
<30 minutes	6802 (82.9)	9399 (61.5)
Missing	0	2737 (17.9)

Data are presented as count (percentage) or mean (standard deviation)
NC= North Carolina

[a] Age was measured at Phase 4.

[b] Other racial and ethnic groups include participants who identified as Non-Hispanic Black, American Indian or Alaska Native, Asian or Pacific Islander, Hispanic/Latino of any race, multiracial, or as 'other' race and measured at Phase 1.

[c] Educational attainment, race, and ethnicity were reported at Phase 1.

[d] Other marital status categories included single, divorced or separated, and widowed (measured at Phase 4).

[e] Sleep duration was assessed by asking participants, "How many hours of sleep do you get each night?". Participants could respond with: 'less than 6 hours', '6 hours to 6 hours and 59 minutes', '7 hours to 7 hours and 59 minutes', '8 hours to 8 hours and 59 minutes', and '9 hours or more'.

[f] Daytime sleepiness was assessed by asking participants: "How often do you feel sleepy most of the day?". Participants could respond with: 'never', 'less than one day per month', '1 to 3 days per month', 1 to 2 days per week', '3 to 5 days per week', '6 to 7 days per week'.

[g] Daytime napping was assessed by asking participants, "Do you nap during the day?". Participants could respond with: 'yes', and 'no'.

[h] Napping duration was assessed by asking participants, "How long do you nap?". Participants who reported taking naps were also asked to indicate their napping duration, response was presented as 'less than 30 minutes', and 'more than 30 minutes'.

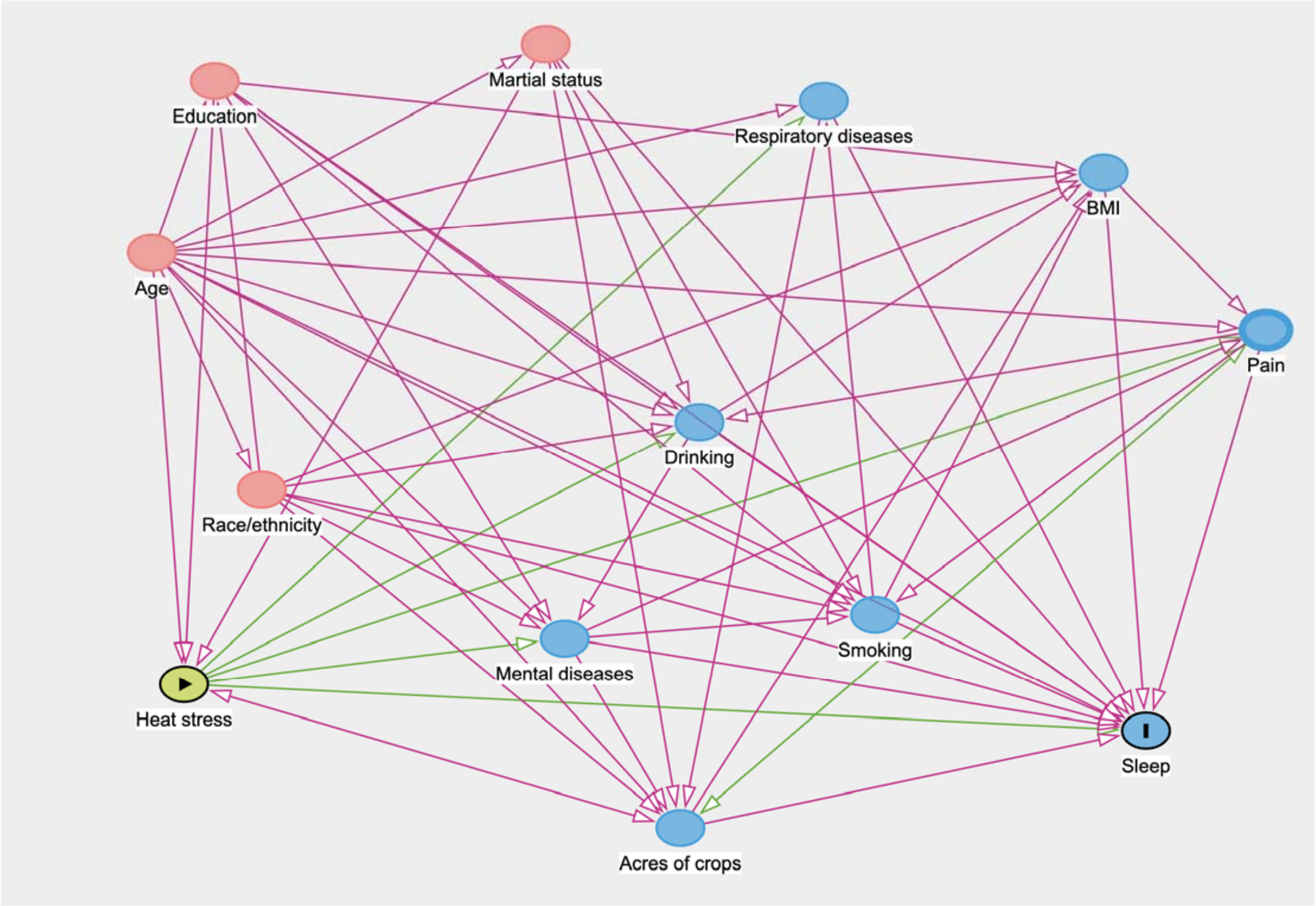

Figure S2. Directed Acyclic Graph (DAG) for the association between heat stress and sleep

medRxiv preprint doi: https://doi.org/10.1101/2025.07.28.25332331; this version posted July 29, 2025. The copyright holder for this preprint (which was not certified by peer review) is the author/funder, who has granted medRxiv a license to display the preprint in perpetuity. This article is a US Government work. It is not subject to copyright under 17 USC 105 and is also made available for use under a CC0 license.

Table S2. The association between each 1 SD increase in WBGT and sleep health among male and female farmers, PR (95%CI).

	PR (95%CI)					
	Iowa (n=6,499)			NC (n=1,854)		
	2-day WBGT	5-day WBGT	7-day WBGT	2-day WBGT	5-day WBGT	7-day WBGT
Sleep duration						
Absolute	1.00 (1.00-1.01)	1.00 (0.99-1.01)	1.01 (1.00-1.01)	1.00 (0.99-1.02)	1.00 (0.99-1.02)	1.00 (0.99-1.02)
Relative	1.00 (1.00-1.01)	1.00 (1.00-1.01)	1.01 (1.00-1.01)	1.00 (0.99-1.02)	1.01 (0.99-1.02)	1.01 (0.99-1.02)
Daytime sleepiness						
Absolute	1.00 (0.99-1.00)	1.00 (0.99-1.00)	1.00 (0.99-1.00)	1.00 (0.98-1.01)	1.00 (0.99-1.01)	1.00 (0.98-1.01)
Relative	0.99 (0.99-1.00)	0.99 (0.99-1.00)	1.00 (0.99-1.00)	1.00 (0.98-1.01)	1.00 (0.99-1.01)	1.00 (0.99-1.01)
Daytime napping						
Absolute	1.00 (1.00-1.01)	1.01 (1.00-1.02)	1.01 (1.00-1.01)	**1.02 (1.01-1.04)**	**1.02 (1.01-1.04)**	**1.02 (1.00-1.03)**
Relative	1.00 (1.00-1.01)	1.01 (1.00-1.01)	1.00 (1.00-1.01)	**1.02 (1.00-1.03)**	1.02 (1.00-1.03)	1.01 (1.00-1.03)
Napping duration						
Absolute	0.99 (0.99-1.00)	1.00 (0.99-1.00)	1.00 (0.99-1.01)	1.00 (0.99-1.02)	1.00 (0.99-1.02)	1.00 (0.99-1.02)
Relative	0.99 (0.99-1.00)	1.00 (0.99-1.00)	1.00 (0.99-1.01)	1.00 (0.99-1.02)	1.00 (0.99-1.01)	1.00 (0.99-1.01)

SD: standard deviation; NC: North Carolina; CI: confidence interval; PR: prevalence ratio; WBGT: Wet Bulb Globe Temperature
All models were adjusted for age, gender, marital status, educational attainment, and race and ethnicity.
Boldface indicates statistical significance at a two-sided p-value of 0.05.

medRxiv preprint doi: https://doi.org/10.1101/2025.07.28.25332331; this version posted July 29, 2025. The copyright holder for this preprint (which was not certified by peer review) is the author/funder, who has granted medRxiv a license to display the preprint in perpetuity. This article is a US Government work. It is not subject to copyright under 17 USC 105 and is also made available for use under a CC0 license.

Table S3. The association between categories of WBGT [a] and sleep health among male and female farmers, PR (95%CI).

	PR (95%CI)					
	Iowa (n=6,499)			NC (n=1,854)		
	2-day WBGT	5-day WBGT	7-day WBGT	2-day WBGT	5-day WBGT	7-day WBGT
Sleep duration						
Moderate risk	**1.03 (1.00-1.07)**	1.03 (0.98-1.08)	1.01 (0.94-1.08)	1.00 (0.96-1.04)	1.00 (0.96-1.03)	1.02 (0.98-1.05)
High risk	1.05 (0.91-1.22)	1.04 (0.87-1.24)	0.91 (0.65-1.27)	1.00 (0.95-1.05)	1.04 (0.99-1.10)	1.03 (0.97-1.10)
Daytime sleepiness						
Moderate risk	1.01 (0.98-1.03)	0.99 (0.96-1.02)	0.98 (0.94-1.02)	0.99 (0.96-1.02)	0.99 (0.97-1.02)	0.99 (0.96-1.02)
High risk	0.97 (0.89-1.06)	1.00 (0.88-1.13)	1.16 (0.82-1.64)	0.99 (0.96-1.03)	1.02 (0.97-1.06)	0.99 (0.94-1.03)
Daytime napping						
Moderate risk	1.01 (0.98-1.04)	1.02 (0.97-1.07)	1.00 (0.94-1.07)	**1.04 (1.00-1.08)**	1.03 (0.99-1.07)	**1.04 (1.00-1.07)**
High risk	0.95 (0.83-1.10)	1.03 (0.86-1.23)	1.26 (0.95-1.66)	**1.07 (1.02-1.11)**	1.02 (0.97-1.07)	1.02 (0.96-1.08)
Napping duration						
Moderate risk	1.00 (0.97-1.03)	1.00 (0.96-1.05)	1.02 (0.96-1.08)	1.01 (0.97-1.05)	1.01 (0.98-1.05)	1.02 (0.98-1.05)
High risk	**0.86 (0.83-0.89)**	**0.89 (0.85-0.92)**	**0.89 (0.86-0.92)**	1.02 (0.98-1.07)	**0.95 (0.91-1.00)**	0.96 (0.91-1.01)

NC: North Carolina; CI: confidence interval; PR: prevalence ratio; WBGT: Wet Bulb Globe Temperature
All models were adjusted for age, gender, marital status, educational attainment, and race and ethnicity.
Boldface indicates statistical significance at a two-sided p-value of 0.05.
[a] Low risk indicates that normal activity is recommended: WBGT <78.8 °F for Iowa and <82.1 °F for NC; moderate risk indicates that planning intense or prolonged activity with discretion is recommended: WBGT 78.8-83.7 °F for Iowa and 82.1-86.0 °F for NC, and high risk indicates that limited or cancelling outdoor activity is recommended: WBGT >83.7 °F for Iowa and >86.0 °F for NC.

medRxiv preprint doi: https://doi.org/10.1101/2025.07.28.25332331; this version posted July 29, 2025. The copyright holder for this preprint (which was not certified by peer review) is the author/funder, who has granted medRxiv a license to display the preprint in perpetuity. This article is a US Government work. It is not subject to copyright under 17 USC 105 and is also made available for use under a CC0 license.

Table S4. The association between each 1 SD increase in WBGT and sleep health among farmers after changing the cut points for sleep duration (<8 hours vs. ≥ 8 hours) and daytime napping (≥1 hour vs. <1 hour), PR (95%CI).

	PR (95%CI)					
	Iowa (n=6,403)			NC (n=1,800)		
	2-day WBGT	5-day WBGT	7-day WBGT	2-day WBGT	5-day WBGT	7-day WBGT
Sleep duration						
Absolute	1.01 (1.00-1.02)	1.01 (1.00-1.02)	1.01 (1.00-1.02)	0.99 (0.96-1.01)	0.99 (0.97-1.01)	0.99 (0.96-1.01)
Relative	1.01 (0.99-1.02)	1.01 (0.99-1.02)	1.01 (1.00-1.02)	0.99 (0.97-1.02)	0.99 (0.97-1.01)	0.99 (0.97-1.01)
Daytime napping						
Absolute	0.90 (0.76-1.08)	0.91 (0.75-1.10)	0.93 (0.77-1.13)	0.88 (0.64-1.21)	0.95 (0.70-1.29)	0.94 (0.69-1.27)
Relative	0.88 (0.74-1.04)	0.88 (0.73-1.05)	0.90 (0.75-1.08)	0.91 (0.67-1.26)	1.01 (0.74-1.37)	0.99 (0.72-1.37)

SD: standard deviation; NC: North Carolina; CI: confidence interval; PR: prevalence ratio; WBGT: Wet Bulb Globe Temperature
All models were adjusted for age, marital status, educational attainment, and race and ethnicity.
Boldface indicates statistical significance at a two-sided p-value of 0.05.

medRxiv preprint doi: https://doi.org/10.1101/2025.07.28.25332331; this version posted July 29, 2025. The copyright holder for this preprint (which was not certified by peer review) is the author/funder, who has granted medRxiv a license to display the preprint in perpetuity. This article is a US Government work. It is not subject to copyright under 17 USC 105 and is also made available for use under a CC0 license.

Table S5. The association between categories of WBGT [a] and sleep health among farmers after changing the cut points for sleep duration (<8 hours vs. ≥ 8 hours) and daytime napping (≥1 hour vs. <1 hour), PR (95%CI).

	PR (95%CI)					
	Iowa (n=6,403)			NC (n=1,800)		
	2-day WBGT	5-day WBGT	7-day WBGT	2-day WBGT	5-day WBGT	7-day WBGT
Sleep duration						
Moderate risk	**1.06 (1.01-1.10)**	1.04 (0.98-1.11)	1.04 (0.96-1.13)	0.99 (0.93-1.05)	0.98 (0.93-1.04)	1.00 (0.95-1.06)
High risk	0.90 (0.70-1.18)	0.88 (0.64-1.21)	0.92 (0.53-1.59)	0.97 (0.91-1.05)	1.00 (0.93-1.08)	0.98 (0.89-1.07)
Daytime napping						
Moderate risk	1.01 (0.51-2.01)	0.97 (0.31-3.04)	1.15 (0.29-4.62)	0.72 (0.31-1.69)	1.07 (0.52-2.20)	1.06 (0.53-2.12)
High risk	-*	-*	-*	0.90 (0.34-2.38)	0.51 (0.12-2.15)	0.67 (0.16-2.80)

SD: standard deviation; NC: North Carolina; CI: confidence interval; PR: prevalence ratio; WBGT: Wet Bulb Globe Temperature
[a] Low risk indicates that normal activity is recommended: WBGT <78.8 °F for Iowa and <82.1 °F for NC; moderate risk indicates that planning intense or prolonged activity with discretion is recommended: WBGT 78.8-83.7 °F for Iowa and 82.1-86.0 °F for NC, and high risk indicates that limited or cancelling outdoor activity is recommended: WBGT >83.7 °F for Iowa and >86.0 °F for NC.
All models were adjusted for age, marital status, educational attainment, and race and ethnicity.
Boldface indicates statistical significance at a two-sided p-value of 0.05.
* Not estimable due to small sample size.

medRxiv preprint doi: https://doi.org/10.1101/2025.07.28.25332331; this version posted July 29, 2025. The copyright holder for this preprint (which was not certified by peer review) is the author/funder, who has granted medRxiv a license to display the preprint in perpetuity. This article is a US Government work. It is not subject to copyright under 17 USC 105 and is also made available for use under a CC0 license.

Table S6. The association between each 1 SD increase in relative heat stress and sleep health among farmers after changing the thresholds for relative WBGT, PR (95%CI).

	PR (95%CI)					
	Iowa (n=6,403)			NC (n=1,800)		
	2-day WBGT	5-day WBGT	7-day WBGT	2-day WBGT	5-day WBGT	7-day WBGT
Sleep duration						
90th percentile	1.01 (1.00-1.01)	1.01 (1.00-1.01)	1.01 (1.00-1.02)	1.01 (0.99-1.02)	1.01 (0.99-1.02)	1.01 (0.99-1.02)
95th percentile	1.01 (1.00-1.02)	1.01 (1.00-1.01)	1.01 (1.00-1.02)	1.01 (0.99-1.02)	1.01 (0.99-1.02)	1.01 (0.99-1.02)
Daytime sleepiness						
90th percentile	0.99 (0.99-1.00)	**0.99 (0.99-1.00)**	0.99 (0.99-1.00)	1.00 (0.98-1.01)	1.00 (0.99-1.01)	1.00 (0.98-1.01)
95th percentile	0.99 (0.99-1.00)	0.99 (0.99-1.00)	1.00 (0.99-1.00)	1.00 (0.98-1.01)	1.00 (0.99-1.01)	1.00 (0.98-1.01)
Daytime napping						
90th percentile	1.00 (1.00-1.01)	1.01 (1.00-1.01)	1.00 (1.00-1.01)	**1.02 (1.01-1.04)**	**1.02 (1.00-1.03)**	1.01 (1.00-1.03)
95th percentile	1.00 (1.00-1.01)	1.01 (1.00-1.01)	1.00 (1.00-1.01)	**1.02 (1.00-1.03)**	1.01 (1.00-1.03)	1.01 (0.99-1.02)
Napping duration						
90th percentile	**0.99 (0.98-1.00)**	0.99 (0.99-1.00)	1.00 (0.99-1.00)	1.00 (0.99-1.02)	1.00 (0.99-1.02)	1.00 (0.99-1.01)
95th percentile	**0.99 (0.98-1.00)**	0.99 (0.99-1.00)	1.00 (0.99-1.00)	1.00 (0.99-1.02)	1.00 (0.98-1.01)	1.00 (0.98-1.01)

SD: standard deviation; NC: North Carolina; CI: confidence interval; PR: prevalence ratio; WBGT: Wet Bulb Globe Temperature
All models were adjusted for age, marital status, educational attainment, and race and ethnicity.
Boldface indicates statistical significance at a two-sided p-value of 0.05.

medRxiv preprint doi: https://doi.org/10.1101/2025.07.28.25332331; this version posted July 29, 2025. The copyright holder for this preprint (which was not certified by peer review) is the author/funder, who has granted medRxiv a license to display the preprint in perpetuity. This article is a US Government work. It is not subject to copyright under 17 USC 105 and is also made available for use under a CC0 license.

Table S7. The association between each 1 SD increase in WBGT and sleep health among male farmers after adjusting for sleep duration or daytime napping, PR (95%CI).

	PR (95%CI)					
	Iowa (n=6,403)			NC (n=1,800)		
	2-day WBGT	5-day WBGT	7-day WBGT	2-day WBGT	5-day WBGT	7-day WBGT
Sleep duration [a]						
Absolute	1.00 (1.00-1.01)	1.00 (1.00-1.01)	1.01 (1.00-1.02)	1.00 (0.99-1.02)	1.01 (0.99-1.02)	1.01 (0.99-1.02)
Relative	1.01 (1.00-1.01)	1.01 (1.00-1.01)	1.01 (1.00-1.02)	1.00 (0.99-1.02)	1.01 (0.99-1.02)	1.01 (0.99-1.02)
Daytime napping [b]						
Absolute	1.00 (1.00-1.01)	1.01 (1.00-1.02)	1.01 (1.00-1.01)	**1.02 (1.01-1.04)**	**1.02 (1.01-1.04)**	**1.02 (1.00-1.03)**
Relative	1.00 (1.00-1.01)	1.01 (1.00-1.01)	1.00 (1.00-1.01)	**1.02 (1.00-1.04)**	1.01 (1.00-1.03)	1.01 (1.00-1.03)

SD: standard deviation; NC: North Carolina; CI: confidence interval; PR: prevalence ratio; WBGT: Wet Bulb Globe Temperature
[a] Models were adjusted for age, gender, marital status, educational attainment, race and ethnicity, and daytime napping.
[b] Models were adjusted for age, gender, marital status, educational attainment, race and ethnicity, and sleep duration.
Boldface indicates statistical significance at a two-sided p-value of 0.05.

medRxiv preprint doi: https://doi.org/10.1101/2025.07.28.25332331; this version posted July 29, 2025. The copyright holder for this preprint (which was not certified by peer review) is the author/funder, who has granted medRxiv a license to display the preprint in perpetuity. This article is a US Government work. It is not subject to copyright under 17 USC 105 and is also made available for use under a CC0 license.
